# Photo-activated polymerization inhibition process in photoinitiator systems for high-throughput 3D nanoprinting

**DOI:** 10.1515/nanoph-2022-0611

**Published:** 2023-01-10

**Authors:** Paul Somers, Zihao Liang, Teng Chi, Jason E. Johnson, Liang Pan, Bryan W. Boudouris, Xianfan Xu

**Affiliations:** School of Mechanical Engineering and Birck Nanotechnology Center, Purdue University, West Lafayette, IN 47907, USA; Charles D. Davidson School of Chemical Engineering, Purdue University, West Lafayette, IN 47907, USA; Department of Chemistry, Purdue University, West Lafayette, IN 47907, USA; Charles D. Davidson School of Chemical Engineering and Department of Chemistry, Purdue University, West Lafayette, IN 47907, USA

**Keywords:** 3D nanoprinting, photoinhibition, projection multiphoton lithography, triplet absorption

## Abstract

The systems for multiphoton 3D nanoprinting are rapidly increasing in print speed for larger throughput and scale, unfortunately without also improvement in resolution. Separately, the process of photoinhibition lithography has been demonstrated to enhance the resolution of multiphoton printing through the introduction of a secondary laser source. The photo-chemical dynamics and interactions for achieving photoinhibition in the various multiphoton photoinitiator systems are complex and still not well understood. Here, we examine the photoinhibition process of the common photoinitiator 7-diethylamino 3-thenoylcoumarin (DETC) with inhibition lasers near or at the multiphoton printing laser wavelength in typical low peak intensity, high repetition rate 3D nanoprinting processes. We demonstrate the clear inhibition of the polymerization process consistent with a triplet absorption deactivation mechanism for a DETC photoresist as well as show inhibition for several other photoresist systems. Additionally, we explore options to recover the photoinhibition process when printing with high intensity, low repetition rate lasers. Finally, we demonstrate photoinhibition in a projection multiphoton printing system. This investigation of photoinhibition lithography with common photoinitiators elucidates the possibility for photoinhibition occurring in many resist systems with typical high repetition rate multiphoton printing lasers as well as for high-speed projection multiphoton printing.

## 1 Introduction

The arena of 3D nanoprinting with multiphoton lithography has experienced drastic growth recently due to the plethora of new printing processes designed to increase the speed and throughput of the process. Through a variety of techniques including multiple beams [1, 2], holography [3], and projection [4, 5], feasible high-speed 3D multiphoton lithography has been demonstrated. At the same time, there has been a desire to increase the resolution of the printing. The nonlinear absorption processes of multiphoton lithography when combined with the photopolymerization process lead to printed features and resolution below the traditional diffraction limit for the wavelength of light used (typically ∼800 nm). The resolution of this printing process is not infinite and reaches a limit typically attributed to the “proximity” and “memory” effect of the photoresist and the requirement of a localized exposure dose large enough for the polymerized region to survive the development process [6]. Several techniques have been implemented to circumvent this resolution limit such as post print pyrolysis [7] and chemical quenching [8]. Two-color photolithography, or photoinhibition lithography, is another promising method for improving the feature size and resolution of 3D printing [9, 10].

Two-color photolithography takes inspiration from Stimulated Emission Depletion (STED) which was introduced to enhance the resolution of fluorescence microscopy [11]. The basic idea is to excite a molecule with one wavelength of light and to prevent it from fluorescing by exposing it with a second wavelength of light. By shaping the second laser source, typically with a donut shape or similar, the overall fluorescing region can be reduced by preventing fluorescence in the outer region exposed with the second laser. Two-color photolithography uses this concept by inducing polymerization with one light source and inhibiting the polymerization with a secondary light source. The second light source can prevent polymerization by changing the state of the initially excited molecule or by interacting with additional molecules in the photoresist that then prevent polymerization. Using the latter, linewidths down to 9 nm have been demonstrated [12]. Two-color photolithography has been achieved with several different photoresist systems with multiple inhibition mechanisms being proposed, including STED [13, 14], triplet absorption (TA) [15–17], and resolution augmentation through photo-induced deactivation (RAPID) [18, 19]. A more comprehensive list of mechanisms can be found elsewhere [10]. Notably, each mechanism has only been proposed to work for a limited set of photoresists, with the possible exception of TA. It would therefore be of interest to further investigate the polymerization inhibition processes of the few known photoinitiators with the hope of finding a photoactivated inhibition pathway that would prove common among a large range of photoinitiators (the idea of which was proposed previously [19]).

The popular photoinitiator 7-diethylamino-3-thenoylcoumarin (DETC) has been proposed to undergo both STED and TA as its polymerization inhibition mechanism [20]. The STED mechanism has been investigated and proven to be efficient for a 532 nm inhibition laser source [13]. That work also showed an inhibition pathway for DETC that exists at longer wavelengths that has been generally attributed to TA followed by reverse intersystem crossing. On the other hand, the TA mechanism has been extensively proven in the photoinitiator isopropyl thioxanthone (ITX) for wavelengths all the way up to 800 nm [17]. In fact, the TA in ITX systems has been proposed to lead to self-deactivation, or polymerization inhibition caused by the printing laser itself. Self-deactivation is attributed to be the cause of an observed effective nonlinearity greater than *N* = 2 (*N* is the number of photons absorbed during the absorption process) found when printing with ITX using an 800 nm fs (typically ∼80 MHz) printing laser. Essentially, the laser is fighting itself to induce polymerization leading to larger required powers than should be expected for a two-photon absorption process. Nonlinearities greater than *N* = 2 resulting from self-deactivation do not result in smaller feature sizes, but rather leads to the opposite effect which is undesirable [17]. The self-deactivation has been simulated in kinetic reaction models which agreed with experimental results [21].

Self-deactivation is not the only source of *N* > 2 observed in multiphoton printing. Particularly, when printing at lower repetition rates the higher peak pulse intensities can induce stronger nonlinear effects such as multiphoton ionization (MPI) [22]. For a molecule that undergoes a process like MPI, a solvated electron is generated. In that case, the traditional radical formation via triplet state does not occur. DETC, among others [23], is also known to exhibit a larger nonlinearity (*N* = 3) than expected for two-photon printing [24]. It is unclear if the nonlinearity is caused by polymerization inhibition (self-deactivation) or by some other pathway such as MPI.

Here we investigate the polymerization inhibition pathway of a DETC containing photoresist at (or near) the printing laser wavelength. We probe for the possibility of short-lived inhibition pathways using pump-probe style inhibition lithography. We explore the possible dependence of the polymerization inhibition pathway on TA from the T_1_ state through the addition of an onium salt coinitiator. We evaluate the polymerization inhibition of DETC and other common photoinitiators at an inhibiting laser wavelength near the printing laser wavelength with a low-cost diode laser. We extend the investigation to printing systems utilizing low repetition rate (5 kHz) fs lasers and evaluate a broad scope of photoresists using a coinitiator to recover the polymerization inhibition effect. We find that while multiple photoinitiators demonstrate inhibition at the printing laser wavelength for high and low repetition rate printing, the inhibition of DETC is the most efficient and promising for use in application. Finally, we demonstrate polymerization inhibition in a multiphoton projection lithography system for high throughput 3D nanoprinting.

## 2 Polymerization inhibition at the printing wavelength

**Figure 1:**
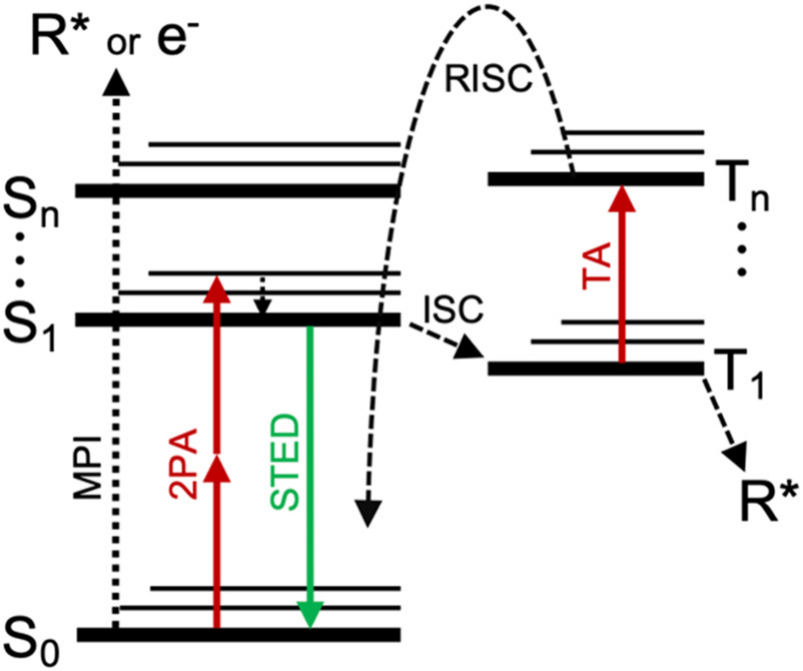
Simplified Jablonski diagram of possible photoinitiator transitions.

The potential molecular pathways for a photoinitiator molecule to undergo using a general energy level diagram are outlined in Figure 1. During normal multiphoton printing the molecule is assumed to undergo two-photon absorption (2PA) from the ground (S_0_) state to reach the S_1_ excited state. It then proceeds by intersystem crossing (ISC) to a triplet state, usually taken to be the T_1_ state. From the T_1_ state a radical (R*) is formed that initiates the polymerization process. Under high intensity radiation it is also possible for the molecule to directly form a highly reactive radical or free electron via a process such as MPI directly from the ground state. Inhibition of the polymerization occurs when the molecule follows a path that does not reach the radical formation. The optical transitions for inhibiting polymerization, STED and TA followed by reverse intersystem crossing (RISC) to a highly vibrationally excited state of S_0_ and subsequent relaxation [17], are also depicted. Polymerization inhibition of DETC via the STED pathway should only be possible for inhibition laser wavelengths that fall within the fluorescence spectra of DETC (indicating that an optical transition from the S_1_ state to the S_0_ ground state exists). Therefore, it is not expected that a laser at the printing wavelength (800 nm) should be able to induce STED in the DETC system (see Supplementary Note 1). The other inhibition mechanism, assumed to be TA, becomes more prominent at longer inhibition wavelengths [13], though it has only been demonstrated up to 642 nm [16]. DETC exhibits a nonzero triplet state absorption at wavelengths up to 800 [16]. Then, like ITX [17], it is possible that polymerization inhibition of DETC via TA can occur at the common wavelength for multiphoton printing (800 nm).

**Figure 2:**
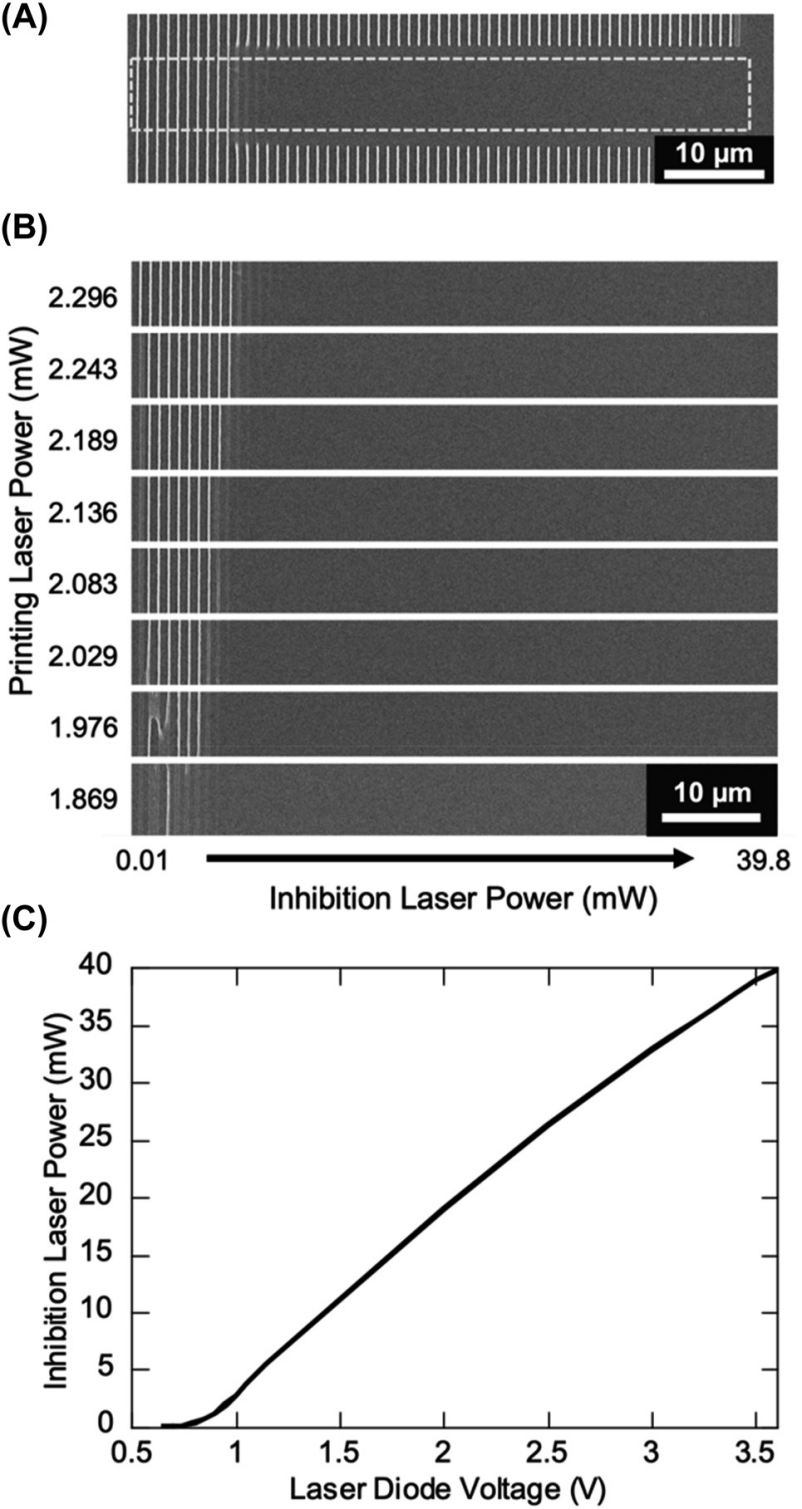
Polymerization inhibition of DETC with continuous wave laser. (A) Example of polymerization inhibition in line printing. Indicated region is what is used for part B of the figure. (B) Line printing of 0.228 mol% DETC in PETA with concurrent illumination by 808 nm CW laser for varying printing laser and CW inhibition laser powers. Inhibition laser diode voltage was increased by 0.05 V for each printed line from left (0.5 V) to right (3.6 V). (C) Inhibition laser power as a function of supplied laser diode voltage.

To test this, an 808 nm CW diode laser is introduced into a typical 80 MHz multiphoton lithography system. The 808 nm laser beam diameter is kept small before the focusing objective to result in a beam spot at least 2 times larger than, ideally, the diffraction limited printing laser spot. The two beams are overlapped spatially within the resist at the print surface. The photoresist for testing was composed of 0.228 mol% (equivalent to 0.25 wt%) of DETC in PETA. For the test, 40 µm long lines separated by 1 µm were printed at 100 μm s^−1^ with the 808 nm laser being turned on for only 10 µm in the middle of fabricating each line (Figure 2A). The region in which both lasers are on at the same time is shown in Figure 2B, where each row of lines is a constant printing laser power. Both the printing laser and the 808 nm laser powers were varied. The 808 nm diode laser was turned on with increasing voltage by 0.05 V steps for each printed line which corresponded to an increased diode laser power for each line. The relationship of diode power to voltage input was not linear, therefore the voltage to power relationship of the 808 nm laser is plotted in Figure 2C for easier visualization of the inhibition power used for each printed line.

It is immediately evident that the polymerization can be inhibited with concurrent irradiation of 808 nm CW laser. In fact, the inhibition effect is very efficient as the polymerization can be completely turned off with ∼5 mW of 808 nm laser or less for a range of printing powers above the printing threshold. This is a promising result for applying to resolution enhancing print schemes (see Supplementary Note 2). It is most likely that the inhibition is a result of absorption of the 808 nm laser while DETC is in the triplet state. The lifetime of the triplet state of DETC is ∼2.3 µs in PETA [16]. The duration of the polymerization inhibition that was observed to emerge using longer wavelength depletion lasers (tested up to 600 nm) is estimated to be of similar order but shorter, less than 1 µs [13]. Assuming the depletion near 600 nm is TA, the difference in triplet state lifetime and measured inhibition duration could be explained by the need for inhibition to occur earlier in the life of the triplet state before radicals can form to initiate polymerization. Nonetheless, as polymerization inhibition of DETC is achieved with 808 nm CW exposure, it is likely that the resist is undergoing the same self-deactivation that is observed in ITX since the lifetime of the triplet state is significantly longer than the pulse-to-pulse separation in the 80 MHz printing laser (12.5 ns). To investigate any interactions with the inhibition laser that occur on timescales shorter than the pulse-to-pulse separation, time-resolved inhibition lithography experiments are performed.

## 3 Pump-probe inhibition lithography

While photoinitiators are generally assumed to undergo intersystem crossing from S_1_ to T_1_, which is usually taken to be the long-lived state that transitions such as TA and radical formation occur from, there is also the possibility of an initial intersystem crossing from S_1_ to T_n_ where T_n_ is a higher triplet state. This would likely be a less stable state resulting in a fast relaxation from that T_n_ state to the T_1_ state. To probe short-lived triplet states before the T_1_ state we perform pump-probe style lithography experiments.

**Figure 3:**
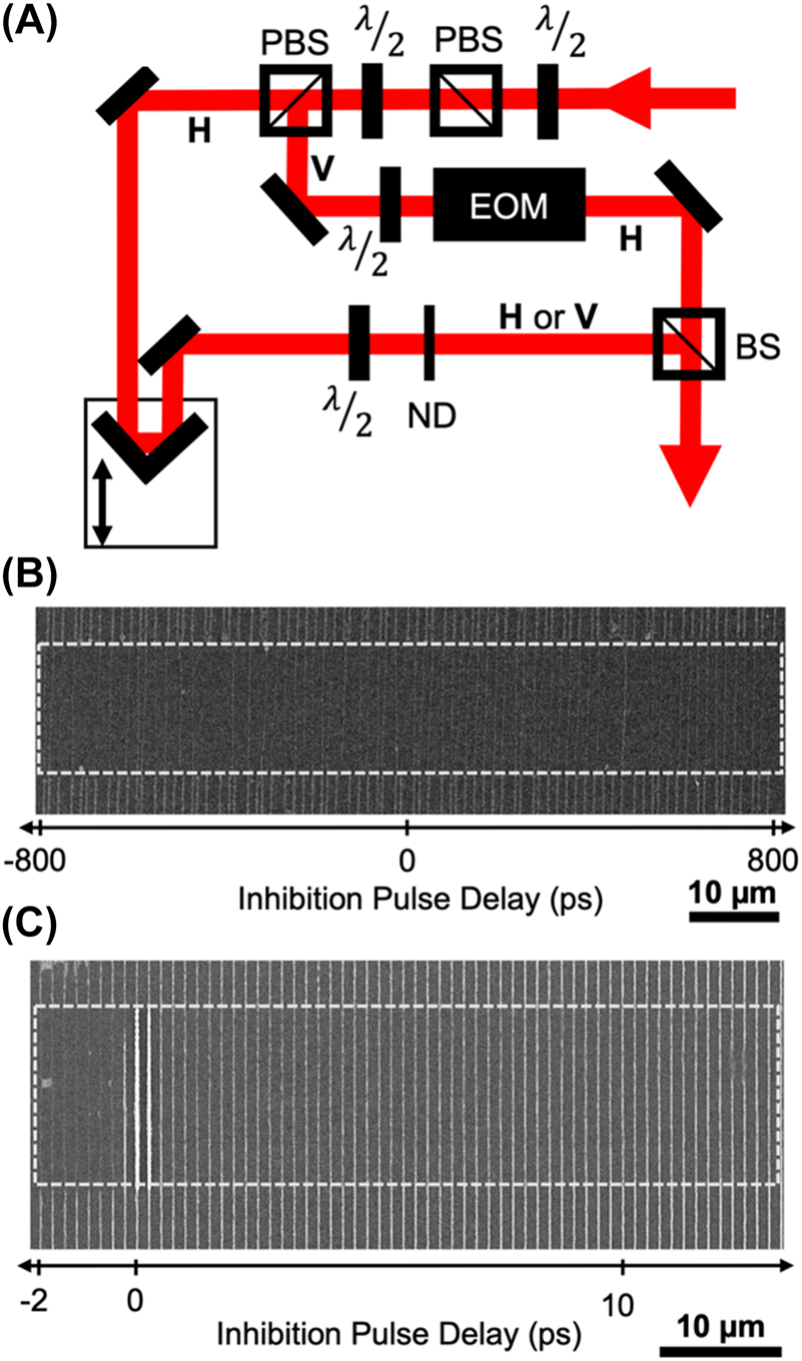
Pump-probe polymerization inhibition of DETC. (A) Schematic of pump-probe lithography system. The polarization state of the beam is indicated as horizontal (H) or vertical (V) relative to the optical table. BS, nonpolarizing beamsplitter; PBS, polarizing beamsplitter; λ/2, half waveplate; ND, neutral density filter; EOM, electro-optic modulator. (B) Lithography results for varying delays in 20 ps steps between the printing pulse and probe or inhibition pulse. Printing laser power was 1.42 mW and probe laser power was 0.885 mW. (C) Lithography results for varying delays in 0.25 ps steps between the printing pulse and probe or inhibition pulse. Printing laser power was 3.06 mW and probe laser power was 1.58 mW. For parts B and C printing was done at 100 μm s^−1^ and the probe, or inhibition laser, was only on during the center part of the figures indicated by the white boxes.

The femtosecond 80 MHz printing laser is split into two beams after a prism pulse compressor. One beam (probe or inhibition beam) is delayed relative to the other (pump or print beam) and the beams are recombined after the printing laser path has passed through an electro-optic modulator (EOM) (Figure 3A). According to the EOM manufacturer documentation, the EOM is expected to contribute >470 fs to the pulse width. As the prism compressor is adjusted to optimize the pulse width of the printing laser arm at the sample, the delayed inhibition arm is stretched by that amount instead, resulting in an inhibition pulse width estimated to be close to 1 ps. No polymerization was observed when using only the probe (inhibition) arm for printing at the same average power as when using the pump (print) arm. For the experiment, 60 µm long lines were printed at a 100 μm s^−1^ speed with the delayed beam only turned on (controlled by mechanical shutter) in the center 20 µm of the printing. The delay between pulses was stepped in increments of 20 ps from −800 ps to 800 ps with 1 µm spacing between the line printed for each time step. The printing laser power was 1.42 mW and probe laser power was 0.885 mW. This probe power was chosen so that there would be no contribution to polymerization while still contributing strongly to any inhibition pathways. The results are shown in Figure 3B. There is inhibition of polymerization for all time delays and no clear time dependent process is observed. Since the polymerization is inhibited even for negative pulse delays (probe pulse arriving before printing pulse) the probe pulse is interacting with a long-lived state (>12.5 ns) like the T_1_ state, as is consistent for TA.

To investigate the existence of any molecular pathways with lifetimes less than the 20 ps step sizes taken in Figure 3B, a pump-probe lithography experiment was done with shorter (0.25 ps) steps in pulse delay (Figure 3C). A clear difference is observed in the effectiveness of the polymerization inhibition for probe pulses arriving before versus after the printing pulse. When the pulses arrive at about the same time (within the pulse duration of the inhibition pulse) constructive interference leads to very high intensities and strong absorption resulting in the bright, significantly more polymerized lines at/near the 0 pulse delay. Printing is almost completely inhibited (similarly to Figure 3B) when the inhibition pulse arrives first. However, the inhibition is not as effective for at least the first 10 ps and more of the inhibition pulse arriving after the print pulse. The results of Figure 3B and C suggest there is a short (∼10 ps) molecular state after excitation that the inhibition probe does not inhibit polymerization. This could be the duration which most of the excited molecules are in the S_1_ state which does not provide a possible optical transition for inhibiting polymerization at 800 nm (inhibition pulse wavelength).

**Figure 4:**
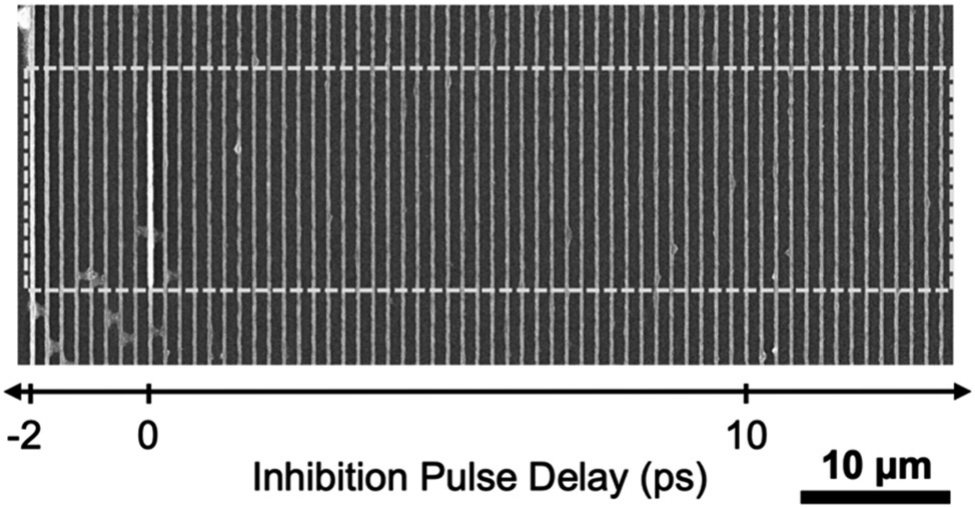
Pump-probe lithography of DETC photoresist containing 0.461% DPIHFP as coinitiator. The inhibition probe beam was turned on during the central region indicated, though no inhibition of printing was observed. Printing laser power was 2.14 mW and the probe laser power was 0.789 mW. Lines were printed at 100 μm s^−1^.

The observed nonlinearity of DETC of *N* greater than 2 has been suggested by a preprint (at time of submission of this work) article [25] to originate from an interaction in a triplet state and was explored by testing the addition of two different types of coinitiators to the photoresist [25]. To further investigate the role TA absorption of 800 nm in DETC plays in contributing to the polymerization inhibition effect, we carried out a similar pump-probe style lithography described above, adding an onium salt coinitiator, diphenyliodonium hexafluorophosphate (DPIHFP) [25], to the photoresist. The photoresist was composed of 0.228 mol% DETC and 0.461 mol% DPIHFP in PETA. As a result of the coinitiator, the writing threshold was observed to be reduced. However, for testing the printing laser power was 2.14 mW and the probe laser power was 0.789 mW in order to show well-structured lines in the new resist. The results are shown in Figure 4. It is clear that the ability to inhibit the polymerization with the probe beam disappears. No inhibition was observed for printing closer to the threshold either (not shown). If the DPIHFP is interacting with the T_1_ state of DETC before any TA can occur, then the loss of the ability to inhibit the polymerization is an indicator that the inhibition pathway of DETC at 800 nm likely relies on TA to work.

## 4 Polymerization inhibition in other photoinitiators

**Figure 5:**
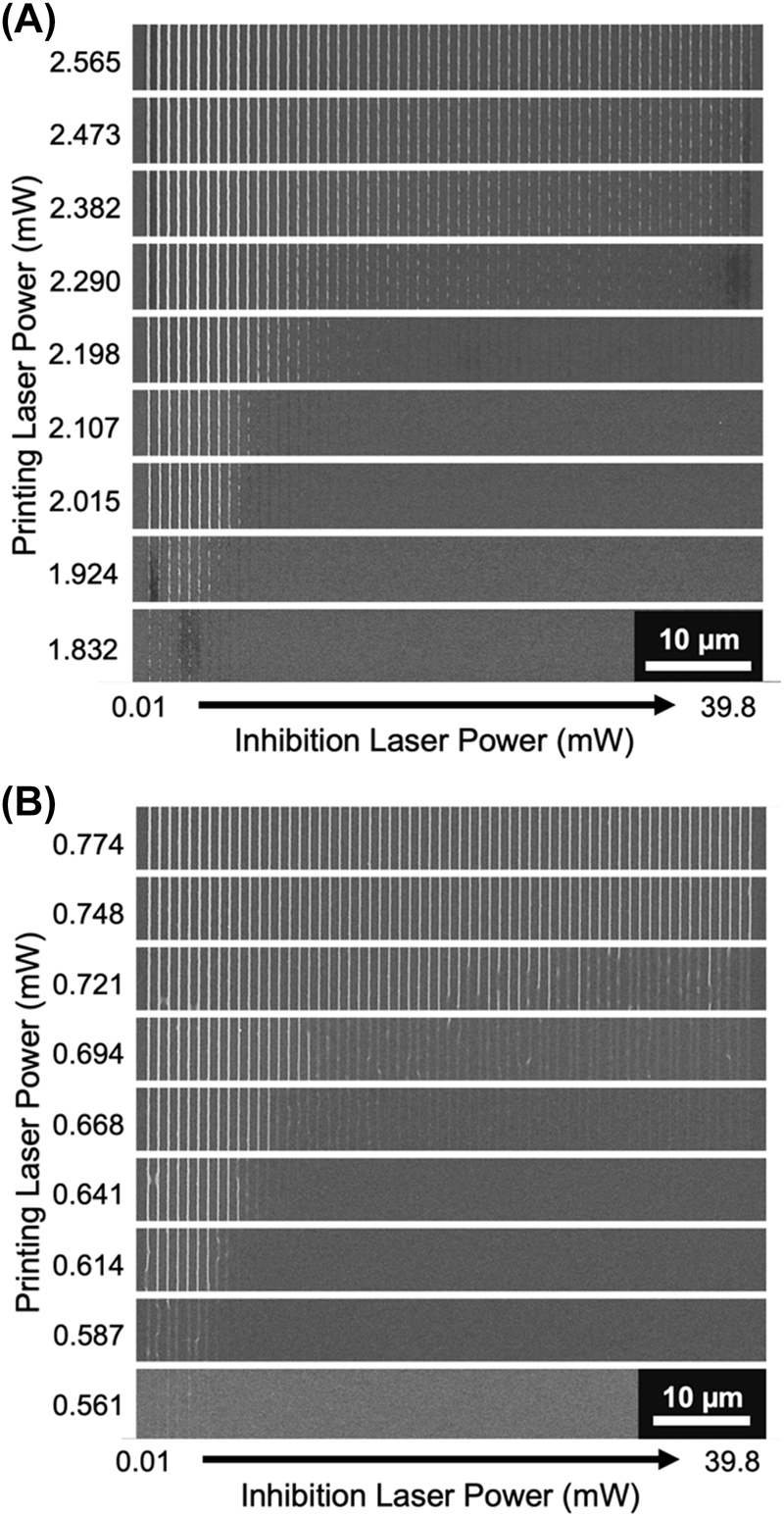
Polymerization inhibition of additional photoinitiators. (A) Line printing using BDEBP in SZ2080 with concurrent illumination by 808 nm CW laser for varying printing laser and CW inhibition laser powers. (B) Same as in panel A but with 0.39% BBK in PETA as the photoresist.

The TA polymerization inhibition pathway has been proposed as a characteristic that could be common to many multiphoton photoinitiators and, indeed, it could be the pathway responsible for the successful photoinhibition of several photoinitiators including ITX, DETC, and 4,4′-bis(diethylamino)benzophenone (BDEBP) with a 642 nm inhibition laser [16]. Therefore, to explore what other photoinitiators may exhibit polymerization inhibition pathways near the printing laser wavelength, several common photoinitiators (and photoresist) were tested using the 80 MHz printing laser and 808 nm CW inhibiting laser. Similar experiments as performed in Figure 2 were done for each of the following resists. The first photoresist tested contained the photoinitiator BDEBP in the SZ2080 resin previously reported [8]. The results in Figure 5A show that a clear inhibition pathway for this resist at 808 nm exists. The next photoresist tested was a mixture of 1 mol% Irgacure 819 in PETA. Irgacure 819 has been shown to exhibit almost no triplet state absorption at 800 nm and shows the expected *N* = 2 nonlinearity for two-photon photoinitiators [16, 22]. Therefore, unsurprisingly, essentially no polymerization inhibition was observed (results not shown). Finally, the recently introduced photoinitiator BBK [26] was tested using a photoresist composition of 0.39 mol% BBK in PETA. The results in Figure 5B indicate a polymerization inhibition path for BBK, though it clearly is not very efficient because of how quickly it disappears at higher print laser powers. BBK has been observed to be a very efficient photoinitiator [5, 26] and likely benefits from a weak self-deactivation effect in normal multiphoton printing applications. The search here has uncovered several more photoresists (see Supplementary Note 3 for additional photoresins) that exhibit polymerization inhibition near the printing laser wavelength.

## 5 Printing and inhibition with high peak power, low repetition rate lasers

Printing with high peak power, low repetition rate lasers enables high-speed, projection multiphoton lithography. However, to this date, almost all polymerization inhibition testing with multiphoton printing has been carried out with high repetition rate printing lasers (around 80 MHz), with some exception [14]. That work explored the STED effect in DETC with variable repetition rate printing laser all the way down to 1 kHz. The STED effect disappeared at lower repetition rate, attributed to a higher *N* = 4 nonlinear absorption process during printing in which the molecule avoided the S_1_ state before polymerization which was needed for STED. At lower repetition rates, the printing pulses have larger intensity, and that intensity is likely the cause of the higher nonlinearity. The authors showed that adding a coinitiator N-phenylglycine (NPG) to the resist lowered the required laser dose for reaching the writing threshold thereby reducing the laser pulse intensity for printing. As a result, the STED polymerization inhibition effect was recovered at lower repetition rates. We have also explored this approach for recovering the polymerization inhibition effect for several inhibition laser wavelengths with a number of different photoinitiators using a 5 kHz printing laser (see Supplementary Note 4).

While the addition of coinitiators has been clearly demonstrated as a way to recover the polymerization inhibition effect at low repetition rates, it has also been observed to have poorer printing resolution [14]. This is speculated to be due to a less confined region of radical generation and diffusion as the coinitiator may form additional radicals alongside the photoinitiator. This type of problem would be drastically amplified for those high-throughput printing processes that already suffer from strong proximity effects [4, 5, 27]. Additionally, depending on the operation of the coinitiator it could reduce the writing threshold while also destroying the inhibition pathway, as is shown above using DPIHFP. The coinitiator could also reduce the possible inhibition laser choices due to undesirable absorption or other interaction with the inhibition laser such as NPG demonstrates with the 638 nm laser (see Supplementary Note 4). For these reasons, it can be argued that adding coinitiators is not the ideal solution.

Another potential solution is to increase the repetition rate of the kHz amplified laser system. This option has its own limitations. Very high repetition rate systems (MHz) struggle to produce the laser power required for high-throughput printing processes such as multiphoton projection printing [5]. Additionally, thermal accumulation effects [22, 28, 29] and self-deactivation could become an issue. Lasers in the tens to hundreds kHz region could feasibly avoid some of these problems as they would allow more time for dissipation of thermal energy between pulses and the time between each laser pulse is longer than the few µs triplet state lifetime of photoinitiators such as DETC. Changing the laser repetition rate is typically not a simple task, however. Therefore, we search for another method to reduce the printing laser intensity at low repetition rates.

We propose creating a pulse burst out of each laser pulse of a low repetition rate laser. This is different than the pulse bursts previously reported which used an AOM to create bursts of pulses separated by 12.5 ns [29]. With only 12.5 ns separation between each pulse in the burst, self-deactivation via TA can still occur since the pulse separation time is shorter than the lifetime of the T_1_ state, reducing the efficiency of the printing. To avoid any potential TA from the T_1_ state, pulses in each burst must all occur before the photoinitiator molecule reaches the T_1_ state. For DETC, the lifetime of the S_1_ state (which occurs before the T_1_ state) is around 1 ns in PETA [13]. In ethanol solution, the S_1_ lifetime has been determined to be 99 ps [20]. Using such a fast burst of pulses with total burst duration below the S_1_ lifetime would allow reducing the peak intensity of each individual laser pulse while exciting the required amount of photoinitiators in each burst to achieve polymerization. The reduced peak intensities would also recover the polymerization inhibition by making absorption by 2PA more likely than other higher order processes. A similar effect could be achieved by simply stretching the fs laser pulse, however some printing processes such as projection multiphoton printing rely on ultrashort pulses to operate effectively so we avoid this option. A simple method of creating a pulse burst (though space intensive) is to use multiple 50:50 beamsplitters to split and recombine the laser. This is what we do here to create a double pulse (with ∼10 ps separation) before entering the projection printing system (see Supplementary Note 5).

**Figure 6:**
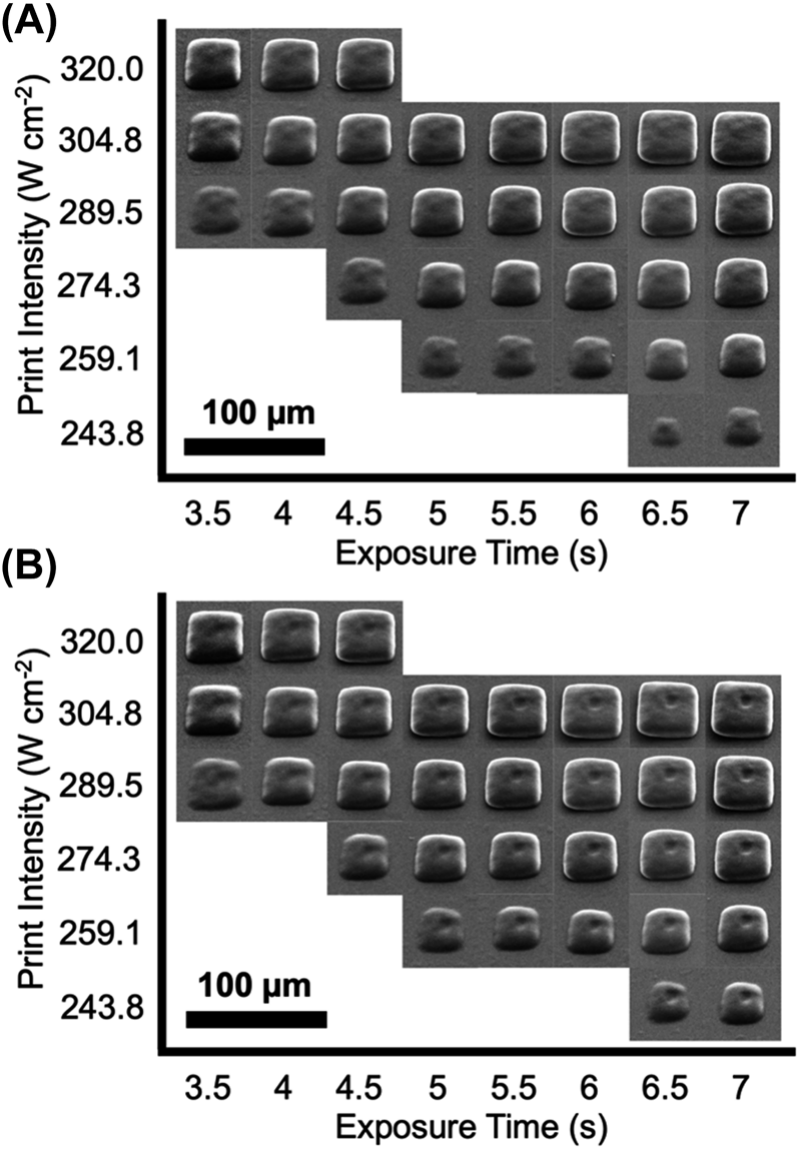
Stationary projection printing using double laser pulse for varying average laser intensity and exposure time. (A) No inhibition laser turned on. (B) 808 nm inhibition laser turned on.

The use of a double pulse with the printing laser is explored for polymerization inhibition in projection multiphoton lithography. A DMD is used to project a square pattern to be printed roughly 43 µm × 43 µm inside the photoresist (0.227 mol% DETC in PETA). A dip-in configuration and printing scheme similar to previously reported [5] is used. To demonstrate inhibiting polymerization, a smaller (10 µm × 10 µm) square pattern formed with the 808 nm CW laser was projected simultaneously (with 18 mW) onto the center of the projection printed square from the opposite direction of the printing laser (see Supplementary Note 6). The printing results for varying print laser average intensities (estimated at the print plane without including objective transmissivity) and exposure times are shown in Figure 6A and B for without and with the inhibition laser turned on, respectively. The tested intensities and exposure times were chosen to explore the near printing threshold conditions. The inhibition effect is observed by the square shaped dimple/hole that can be observed in the middle of the prints in Figure 6B. For comparison, the same experiment was performed using a single laser pulse (Figure S6).

It is evident that as the exposure time gets smaller and print laser intensity gets higher, the inhibition effect is not as prominent. This is expected as the peak laser intensities increase as the exposure time is reduced to meet the required dose for polymerization. At the higher intensities, the nonlinear paths other than 2PA begin to take over, such that processes like TA cannot occur to inhibit the polymerization. Noticeably, the inhibition does not appear to go through the entire printed pattern. This could be because the inhibition laser power was not high enough, though the maximum available power in the setup was used. Alternatively, there is a temporal focusing effect occurring during the print process leading to a change in the printing laser pulse width (and thus intensity) along the height of the printed structure. It could be possible that at the temporal focus the laser intensity is high enough to induce higher nonlinear absorption and away from the temporal focus the absorption changes to predominantly 2PA. If that is the case, the inhibition can be used to at least turn off polymerization in the regions of 2PA. Using a large area inhibition laser exposure, the print layer thickness and resolution could be improved so that polymerization only occurs in the volume of higher nonlinear absorptions (which should be smaller than 2PA regions). An unexpected benefit of the double pulse for projection printing was smoother, more uniform print profiles (compare to Figure S8). This is attributed to an averaging of the laser speckle of the two laser paths.

This is the first evidence of polymerization inhibition implemented into a projection multiphoton printing system to the authors’ knowledge. On the other hand, the timescales of exposure used to achieve noticeable polymerization inhibition here are too long to directly translate into use at the high print speeds regularly utilized in the projection printing systems. A pulse burst with many more pulses than the two used here may provide a solution.

## 6 Conclusions

This work investigated the polymerization inhibition properties of the photoinitiator DETC with inhibition wavelengths at and near the common multiphoton printing laser wavelength of 800 nm using an 80 MHz printing laser. An efficient inhibition pathway was found, and is attributed to be likely due to the commonly suggested TA mechanism, investigated through a combination of pump-probe lithography experiments and demonstration of lost inhibition ability due to expected quenching of the T_1_ state with onium salt-based coinitiator. We further showed that a common polymerization inhibition pathway exists for many photoinitiators such as BDEBP and BBK. The fact that inhibition occurs near the printing wavelength of 800 nm implies self-deactivation may exist in multiple photoresist systems. We also showed that it is critical that the photoinitiator initiates polymerization through the S_1_ to T_1_ pathway, otherwise optical polymerization inhibition cannot occur. Higher nonlinear optical absorptions than two-photon absorption that occur when printing with high intensity, low repetition rate lasers (5 kHz in this work) remove the observed polymerization inhibition effect. By reducing the pulse intensities used during printing, the polymerization inhibition was recovered. Using a two-pulse burst for printing, each pulse with reduced intensity, we demonstrate inhibition lithography with projection multiphoton printing. This work expands the knowledge base and toolset of photoinhibition lithography while also highlighting the opportunities of implementing photoinhibition lithography into high-throughput 3D nanoprinting systems.

## 7 Methods and materials

### 7.1 Materials

Chemicals including methanol, 4-(dibutylamino)benzaldehyde, 4-methylcyclohexanone, potassium hydroxide, n-phenylglycine (NPG), ITX, phenylbis(2,4,6-trimethylbenzoyl) phosphine oxide (Irgacure 819) and pentaerythritol triacrylate (PETA, #246794) were purchased from Sigma–Aldrich. DETC was purchased from J & K Scientific. IP-S and IP-Dip were purchased from Nanoscribe. All above chemicals were used as received. Any required synthesis of photoinitiators or photoresins is detailed in Supplementary Note 7.

### 7.2 High repetition rate experiments

An 80 MHz fs oscillator (Coherent Micra-10) was used for the high repetition rate printing experiments. The printing system was a modified version of a previously reported setup [30]. The center wavelength and bandwidth were 800 nm and 30 nm, respectively, with a full-width at half-maximum (FWHM) pulse width at the sample plane of ∼390 fs. Pulses at the sample were compressed with a home-built double-prism compressor. A half-wave plate and polarizing beamsplitter are used for coarse power control and a mechanical shutter (Uniblitz LS3ZM2) is used for on/off control. The beam is passed through an EOM (ConOptics 350-160c) which provided fine power control. Samples were mounted on an inverted microscope (Nikon Eclipse Ti-U) and translated using a 3-axis nanopositioner (Mad City Labs Nano-LP Series). Photoresists were sandwiched between a microscope slide and coverslip, using tape as a spacer to provide a gap of ∼35 µm. The printing (and inhibition) laser was focused into the resist using oil immersion and a 100 × immersion oil objective lens (Nikon N.A. = 1.49). For pump-probe inhibition experiments, the printing laser was split after the compressor but before the EOM using another half-wave plate and polarizing cube (see Figure 3A). The split beam (probe or inhibiting beam) was then reflected off a retroreflector on a linear motion-controlled stage (Newport MTM250PP.1) which provided delay control. The polarization of the probe beam was then set to match the printing laser polarization using a half-wave plate. A separate mechanical shutter (Uniblitz LS2ZM2) was used to control the probe beam. The beams were recombined using a nonpolarizing 50:50 beamsplitter before the microscope. For continuous wave (CW) inhibition experiments an 808 nm diode laser (Thorlabs LD808-SE500) was introduced through the same 50:50 beamsplitter. Power control of the 808 nm laser was accomplished by voltage supplied to the controller. All printing was controlled via custom LabVIEW programs. Laser powers quoted in this setup are measured at the back entrance of the objective lens through a 6 mm aperture. All printing was done at 100 μm s^−1^ stage speed to print lines unless otherwise specified.

### 7.3 Low repetition rate experiments

A 5 kHz regeneratively amplified femtosecond laser (Coherent Legend II Duo) was used for the low repetition rate experiments. The center wavelength and bandwidth were 800 nm and 27 nm respectively. Pulse width out of the amplifier was estimated to be <50 fs. A half-wave plate and polarizing cube were used for power control. The beam diameter was reduced to ∼6 mm before passing through a mechanical shutter (Uniblitz LS6T2) for on/off control. The beam was then focused through a 100 × immersion oil objective lens (Nikon N.A. = 1.49) to the sample. The objective was operated in a dip-in configuration with the photoresist directly in contact with the objective. Printing substrates were microscope slides and sample positioning was done with a 3-axis air-bearing stage (Aerotech ABL1000 series). For polymerization inhibition testing, three CW lasers (638 nm, Thorlabs L638P200; 532 nm, Spectra-Physics Millenia; 808 nm, Thorlabs LD808-SE500) were introduced into the beam path via dichroic mirrors. The 638 nm and 808 nm diode lasers were controlled by the voltage supplied to their controllers. The 532 nm laser power was controlled using an acousto-optic modulator (AOM, NEOS *N*35085-3). All printing processes were controlled via Aerotech’s NVIEW software. All printing was done at 100 μm s^−1^ stage speed to print lines.

The same amplified laser was used for the projection printing. After the half-wave plate and polarizer cube, the beam was passed through a πShaper (AdlOptica 12_12_TiS_HP) before being incident on a digital micromirror device (DMD, TI DLP4500NIR). The diffracted light pattern was collected with an achromatic doublet (*f* = 150 mm, Thorlabs AC508-150-B) before being imaged into the photoresist through a 100 × objective lens (Nikon N.A. = 1.25 model MRP01902) being used in dip-in configuration. The same air-bearing stage was used for sample positioning. For inhibition experiments, the 808 nm diode laser was focused using an *f* = 150 mm lens through a 100 µm × 100 µm square aperture fabricated on a Au-coated (>100 nm thick) microscope slide using focused ion beam milling. The focusing was done to pass the majority of the beam power through the aperture. After the aperture, the light was collected with an *f* = 200 mm lens before reforming the image through a 10 × objective lens (Nikon N.A. = 0.3) to the print plane of the projection printing system. The 10× objective imaged the 808 nm light from the opposite direction of the projection printing light (printing from above and inhibition from below, see Figure S5).

### 7.4 Photoresist preparation and post-print processing

Photoresist mixtures were created by mixing a photoinitiator (and coinitiator if used) with the monomer PETA and sonicating overnight. All samples were developed in a bath of isopropanol (IPA) for 10–15 min before being rinsed with IPA and dried in ambient conditions. Samples were sputter coated with a Au or Au/Pd mixture (10–20 nm) before being imaged with an SEM (Hitachi S-4800). Typical parameters for imaging were 15 kV and 5 mA.

## Supplementary Material

Supplementary Material Details
