# Supplementary material for: Photo-activated polymerization inhibition process in photoinitiator systems for high-throughput 3D nanoprinting
Source: Nanophotonics. 2023 Jan 10;12(8):1571–80. doi: 10.1515/nanoph-2022-0611 (PMC11501553; doi:10.1515/nanoph-2022-0611)
Supplement: Supplementary file 1 — Supplementary Material Details [file j_nanoph-2022-0611_suppl.pdf]

Supplementary Material

Paul Somers, Zihao Liang, Teng Chi, Jason E. Johnson, Liang Pan, Bryan W. Boudouris, Xianfan Xu*

Photo-activated polymerization inhibition process in photoinitiator systems for high-throughput 3D nanoprinting

***Corresponding author: Xianfan Xu,** School of Mechanical Engineering and Birck Nanotechnology Center, Purdue University, West Lafayette, Indiana 47907, USA, email: xxu@ecn.purdue.edu

Supplementary Note 1: 7-diethylamino 3-thenoylcoumarin (DETC) Spectra

The ultraviolet-visible (UV-Vis) and fluorescence spectra for DETC in the monomer pentaerythritol triacrylate (PETA) is shown in **Figure S1**.

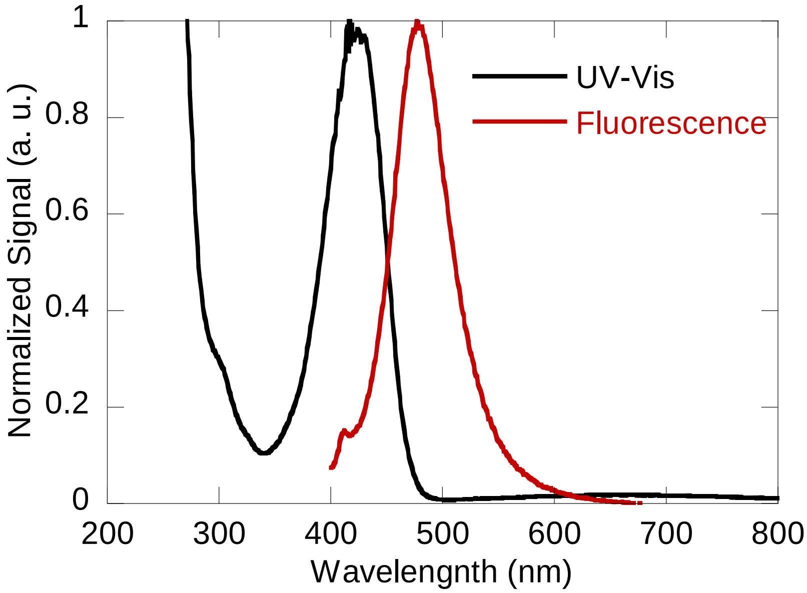

Figure S1. UV-Vis and fluorescence spectra of DETC in PETA.

Supplementary Note 2: Reducing Print Feature Size with Donut Profile

To demonstrate the potential of using photoinhibition with lasers near the printing wavelength to improve printing feature size, lines were printed while using an 800 nm continuous wave (CW) laser with a donut shaped profile as the inhibition laser. For the experiment, an 800 nm fs-laser (80 MHz, Coherent Micra) was used for printing the lines. A Coherent Mira fs-oscillator was operated in CW mode at ~800 nm and used as the inhibition laser. Due to mode-hopping of the Mira in CW mode, there was some instability in the laser power and pointing. A spiral waveplate (Vortex Photonics, V-800-10-1) was added to the CW beam path to create a donut focus profile and then the two beams were combined with a 50:50 beamsplitter. The donut focus was centered on the Gaussian printing laser focus using a microscope camera. A set of 20 lines was printed at 100 µm s^{-1} in a photoresist of 0.228 mol% of DETC in PETA and the results are shown in **Figure S2**. For part of the lines printed both lasers were on and for part of the lines only the printing laser was on (indicated in the figure by the insets). A clear reduction in linewidth is observed when the donut-shaped inhibition laser is on. Printing laser power was 5.083 mW and inhibition laser power was 75.72 mW (15× larger than printing laser power).

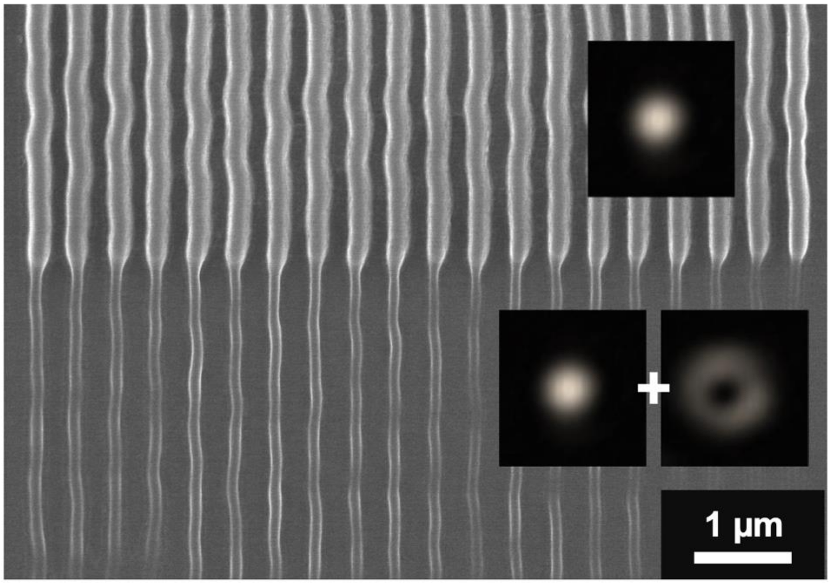

Figure S2. Lines printed using donut shaped inhibition laser. Insets are camera images of the printing (Gaussian) and inhibition (donut) laser foci (not to scale). Only the printing laser is on in the upper half of the figure and both lasers are on in the lower half.

Supplementary Note 3: Polymerization Inhibition Using Nanoscribe Resists

The IP-S photoresist from Nanoscribe was tested in a similar experiment to Figure 5. The exact components of IP-S are proprietary so the photoinitiator is unknown. The results in **Figure S3** show that a clear inhibition pathway for IP-S at 808 nm exists. Similar experiments were performed on IP-Dip resist from Nanoscribe with similar results (results not shown).

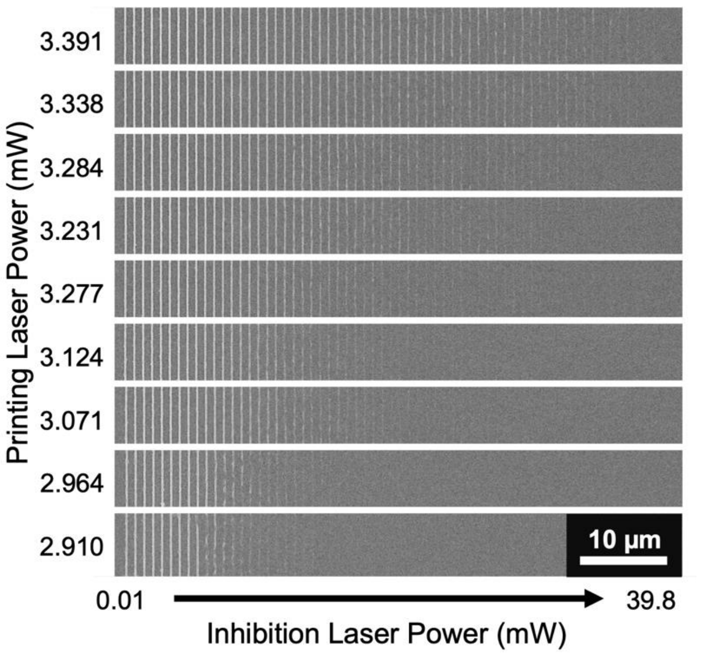

Figure S3. Line printing using IP-S with concurrent illumination by 808 nm CW laser for varying printing laser and CW inhibition laser powers.

Supplementary Note 4: Addition of Coinitiators for Recovering Inhibition

The effect of adding a coinitiator to the photoresist to recover the polymerization inhibition effect when printing with a 5 kHz laser was explored.

Table S1 lists the photoinitiators and their concentration, as well as the varying concentrations of N-phenylglycine (NPG), that were mixed. The writing thresholds of each photoresist were determined for printing lines at 100 µm s^{-1} (**Figure S4**). As a result of the threshold test, not all photoresists were carried forward for inhibition testing. However, inhibition testing was performed on at least one or two photoresists containing each photoinitiator to check if any photoinitiator showed unexpectedly efficient depletion. For the most part, only the photoresists that showed a writing threshold near that of the DETC without coinitiator resist were tested further. To test the polymerization inhibition property three CW inhibition lasers were introduced to the printing laser path and overlapped with the printing laser. The first, 532 nm, laser (G in **Table S1**) did not exhibit polymerization inhibition in any of the tested resists. It was found to contribute to polymerization, especially in the NPG containing resists. The other two lasers, 638 nm and 808 nm (R and X in **Table S1**, respectively), exhibited more success so only the results with those two lasers are shown here.

Table S1. List of photoinitiators with concentration and coinitiator additions that were tested. The inhibition lasers tested for each resist are indicated. G – 532 nm laser, R – 638 nm laser, X – 808 nm laser.

	**0% NPG**	**0.25% NPG**	**0.5% NPG**	**1.0% NPG**
1.0% ITX-p-OCH_3		R, X	R, X	Not Soluble
0.227% DETC	G, R, X	G, R, X	G, R, X	R, X
1.0% ITX			R, X	R, X
1.76% ITX			R, X	
1.75% ITX-nBuT	R, X		R, X	
0.39% BBK	R, X			
0.228% OMe-DETC		R, X		R, X

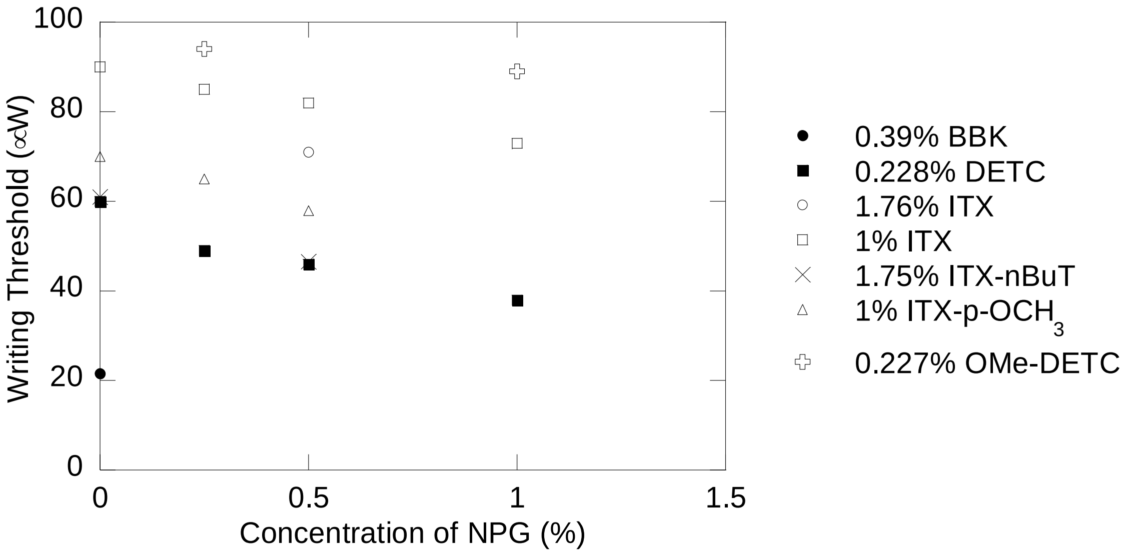

Figure S4. Writing thresholds for different photoinitiator systems with different concentrations of NPG. Writing speed was 100 µm s^{-1}.

To evaluate the efficiency of the polymerization inhibition, the change in the writing threshold under exposure to the inhibition laser was plotted as a function of the depletion laser power for each tested resist. When being exposed with the inhibition laser, the printing laser power required to overcome the inhibition and print a line was compared to the printing laser threshold power without the inhibition laser on. An increased print laser power under those conditions means a positive threshold shift with a larger shift meaning more efficient inhibition. A negative shift means the inhibition laser contributed to the polymerization. The results are shown in **Figure S5**. No resists without coinitiator demonstrated any measurable amount of polymerization inhibition. Most resists with coinitiator exhibited at least some level of inhibition at both wavelengths. At higher powers the 638 nm laser tended to start contributing to polymerization while the 808 nm did not. This may have been due to 638 nm being closer to the absorption region of the photoresist. For those resists that did demonstrate inhibition, increasing NPG concentration improved the efficiency of the inhibition. The ITX photoresists seem to show that 638 nm achieves a more efficient polymerization inhibition than 808 nm. This can be expected because 638 nm is close to the TTA peak for ITX [1]. Most of the shifts were small, but DETC proved noticeably to be the most efficient at inhibiting. The threshold shift appears to plateau, with increasing depletion laser power having no effect (within the range of depletion laser power available during testing). This may be a result of the higher nonlinear process dominating the printing laser absorption at the print laser power corresponding to the maximum threshold shift. Clearly, adding a coinitiator achieved the desired result of reducing peak printing laser intensities and bringing back the inhibition properties of at least some photoinitiators.

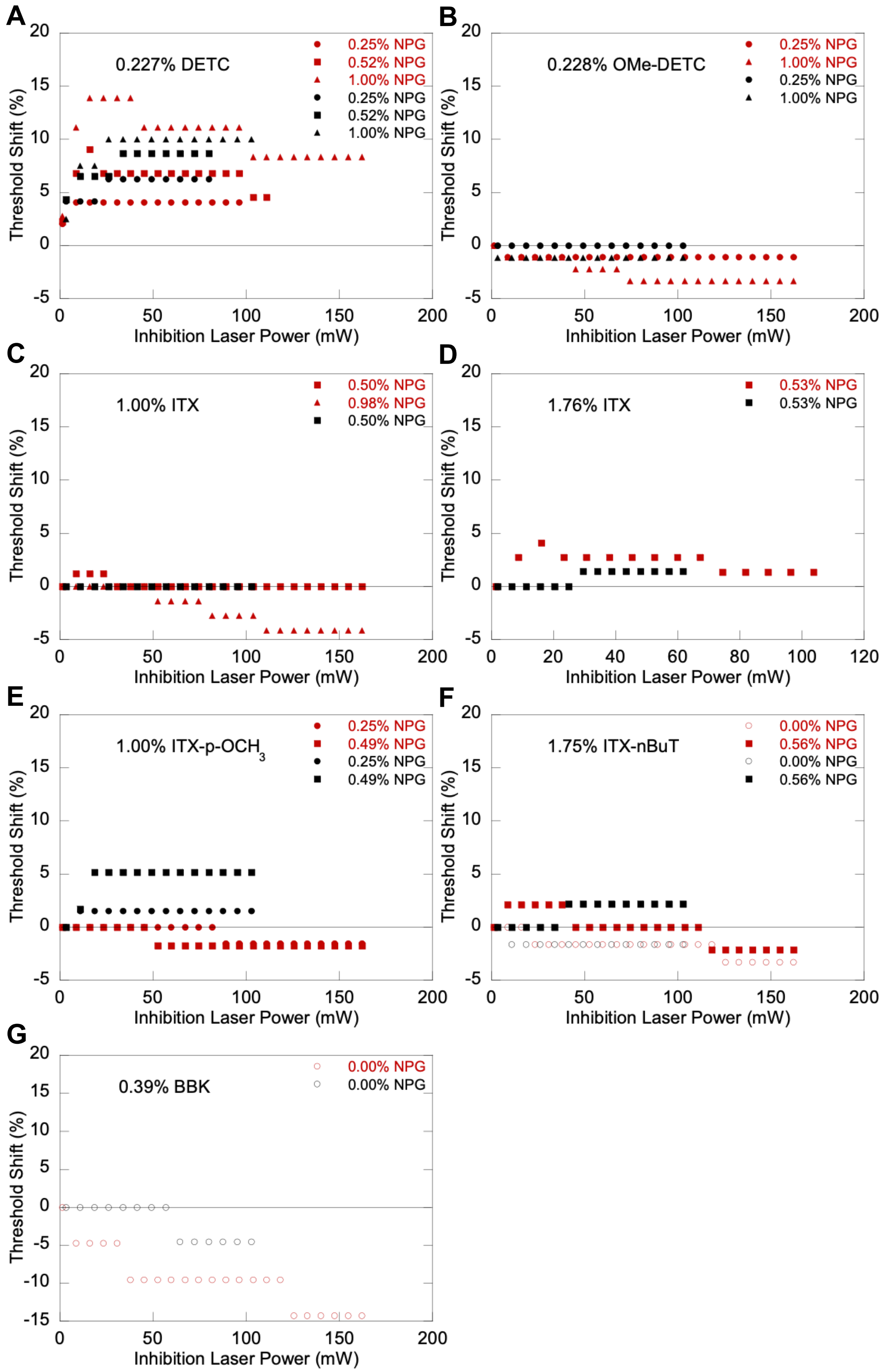

Figure S5. Plots of writing threshold shift for different photoresists. Threshold shift is plotted against varying depletion laser power. 638 nm (red) and 808 nm (black) inhibition lasers were used. Photoinitiator and concentration are indicated, and results are plotted for different additions of NPG coinitiator.

<u>Supplementary Note 5: Making a Double Pulse</u>

A double pulse was generated in a 5 kHz repetition rate laser in order to reduce the peak intensities inside the photoresist in an attempt to make polymerization inhibition more likely to occur. The double pulse was created by using 50:50 beamsplitters to split and recombine the laser with a slight delay (estimated to be <10 ps) between the pulses as shown in **Figure S6**. Due to one arm reflecting off more mirrors (hence more energy losses) the pulses were not equal power with the first pulse in the burst having 62% of the power and the second pulse having 38%.

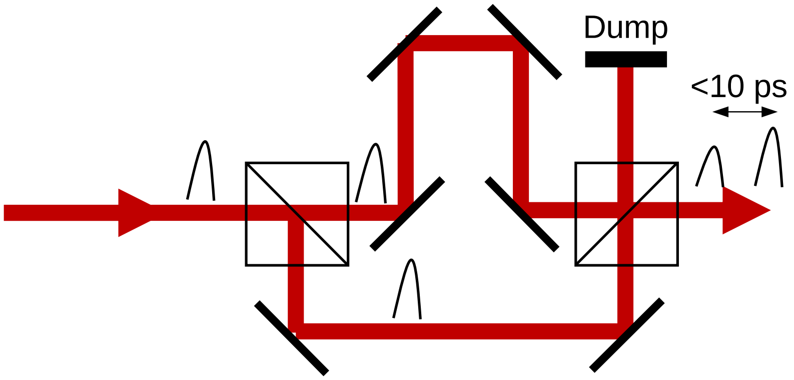

Figure S6. Schematic of double pulse burst generation using 50:50 beamsplitters.

Supplementary Note 6: Projection Printing and Inhibition Without Splitting the Pulses

Similar to Figure 6, projection printing of 43 µm × 43 µm squares was done in a photoresist of 0.227 mol% DETC in PETA. Simultaneously, a 10 µm × 10 µm square pattern was projected using an 808 nm inhibition laser (18 mW at the sample) from the opposite direction as shown in **Figure S7**. The original 5 kHz single laser pulses were used for the projection printing (no double pulse). The results are shown in **Figure S8**. Lower average print intensities were required to meet the printing threshold because each individual laser pulse was higher intensity than the individual laser pulses used in Figure 6.

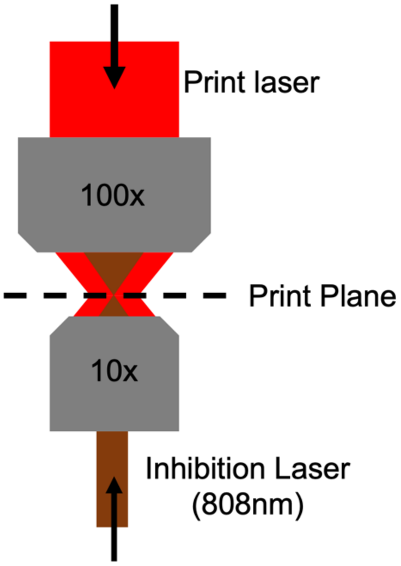

Figure S7. Schematic of projection printing with simultaneous inhibition laser.

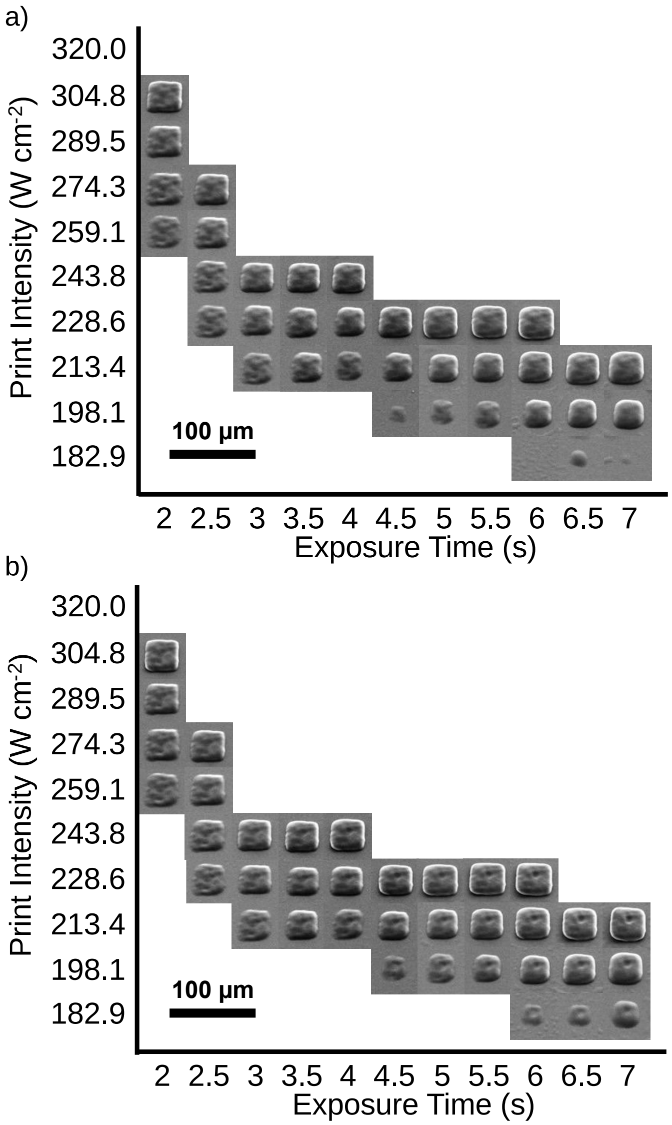

Figure S8. Stationary projection printing using single laser pulse for varying average laser intensity and exposure time. a) No inhibition laser turned on. b) 808 nm inhibition laser turned on.

Supplementary Note 7: Synthesis of Photoinitiators

Synthesis of (2E,6E)-2,6-Bis (4-(dibutylamino) benzylidene)-4-methylcyclohexanone (BBK)
The photoinitiator BBK was synthesized as reported previously [2].

Synthesis of 2-isopropyl-7-(4-methoxyphenyl)-9H-thio-xanthen-9-one (ITX-p-OCH_3)
The photoinitiator ITX-p-OCH_3 was synthesized as reported previously [3].

Synthesis of SZ2080
The photoresin SZ2080 was synthesized as reported previously [4].

Synthesis of 2-(5-butylthiophen-2-yl)-7-isopropyl-9H-thioxanthen-9-one (ITX-nBuT)
In a 25 mL Schlenk tube, 2-bromo-7-isopropyl-9H-thioxanthen-9-one (ITX-Br) (66 mg, 0.2 mmol) and 2-(5-butylthiophen-2-yl)-4,4,5,5-tetramethyl-1,3,2-dioxaborolane (58.5 mg, 0.22 mmol) were dissolved in 2 mL toluene. Then, sodium carbonate (53 mg, 0.5 mmol), Tetrakis(triphenylphosphine)palladium(0) (30 mg, 0.02 mmol) that were dissolved in 1 mL deionized water and 1 mL ethanol were added into the reaction vessel. The oxygen in the vessel was removed through three cycles of freeze-pump-thaw using liquid nitrogen on a Schlenk line. This reaction was stirred for 8 h at 96 ℃. When the reaction finished, the precipitate generated was removed by filtration, washed by ethyl acetate for three times, and the clear solution was concentrated using a rotary evaporator. This crude mixture was purified using flash chromatography on silica gel with hexane: ethyl acetate = 20:1 v/v as the eluent. 43.7 mg of the product was collected as a yellow solid (yield = 56 %). ^{1}H NMR (400 MHz, CDCl3) δ 8.79 (d, J = 2.0 Hz, 1H), 8.49 (s, 1H), 7.79 (dd, J = 8.4, 2.2 Hz, 1H), 7.62 – 7.48 (m, 3H), 7.28 (d, J = 3.8 Hz, 1H), 6.79 (s, 1H), 3.08 (dq, J = 14.0, 7.0 Hz, 1H), 2.85 (t, J = 7.7 Hz, 2H), 1.80 – 1.65 (m, 2H), 1.49 – 1.39 (m, 2H), 1.33 (d, J = 6.8 Hz, 6H), 0.96 (t, J = 7.3 Hz, 3H).

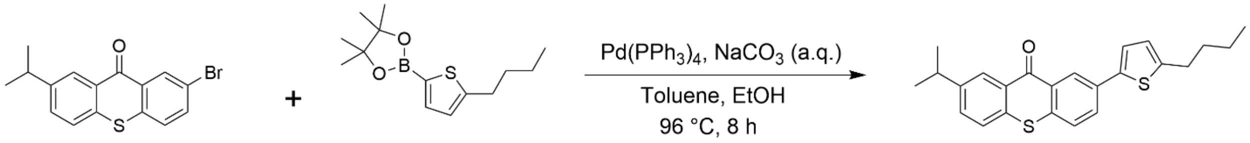

Scheme S1. Synthetic method of 2-(5-butylthiophen-2-yl)-7-isopropyl-9H-thioxanthen-9-one (ITX-nBuT)

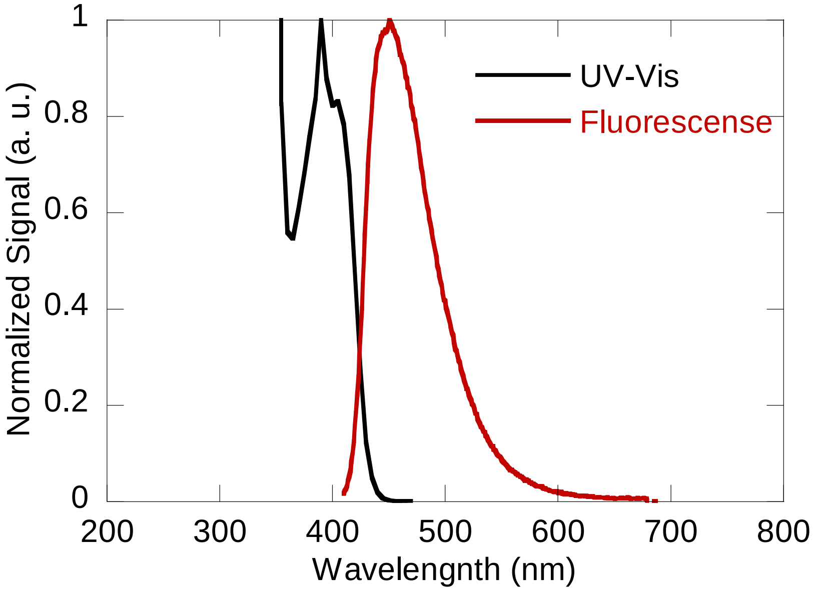

Figure S9. UV-Vis absorption and fluorescence spectra for ITX-nBuT in tetrahydrofuran (THF) solution.

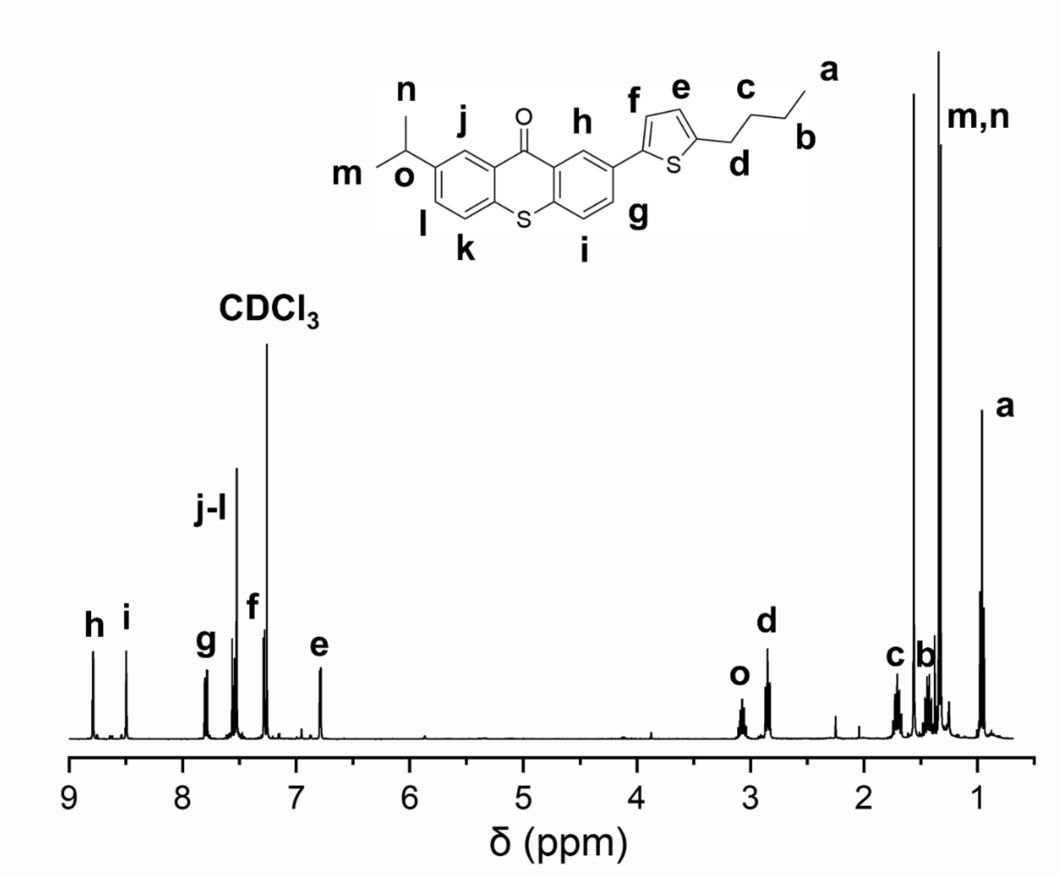

Figure S10. ^{1}H NMR spectra of 2-(5-butylthiophen-2-yl)-7-isopropyl-9H-thioxanthen-9-one (ITX-nBuT). Product was dissolved in deuterated chloroform ($CDCl_3$).

Synthesis of 6-methoxy-3-(thiophene-2-carbonyl)-2H-chromen-2-one (OMe-DETC)

2-hydroxy-5-methoxybenzaldehyde (75 mg, 0.5 mmol), ethyl 3-oxo-3-(thiophen-2-yl)propanoate (100 mg, 0.5 mmol), triethylamine (0.5 mL, 3.6 mmol) were added into a 25 mL Schlenk flask. Then 4 mL toluene was added as the solvent. The reaction vessel was degassed using the freeze-pump-thaw method. Then, the reaction was stirred under 100 °C for 12 h. Then, the reaction was cooled to the room temperature, and white solids precipitated from the solution. The precipitation was collected using on a filter paper and rinsed with hexane for three times to remove the impurities to generate 48 mg of white solid as the product (yield = 33 %). ^{1}H NMR (400 MHz, DMSO) δ 8.38 (s, 1H), 8.16 (dd, J = 4.9, 1.2 Hz, 1H), 7.92 (dd, J = 3.8, 1.2 Hz, 1H), 7.43 (d, J = 9.0 Hz, 1H), 7.38 (d, J = 3.0 Hz, 1H), 7.35 – 7.23 (m, 2H), 3.80 (s, 3H).

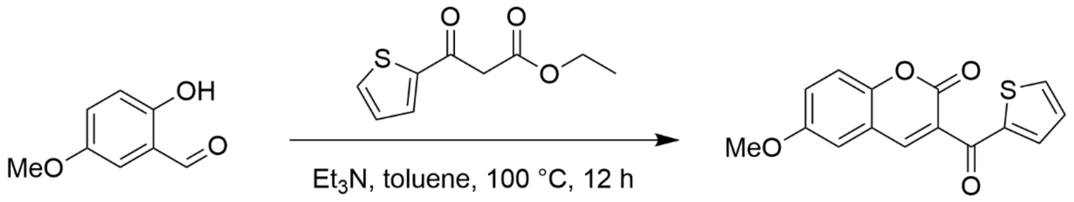

Scheme S2. The synthetic method of 6-methoxy-3-(thiophene-2-carbonyl)-2H-chromen-2-one (OMe-DETC).

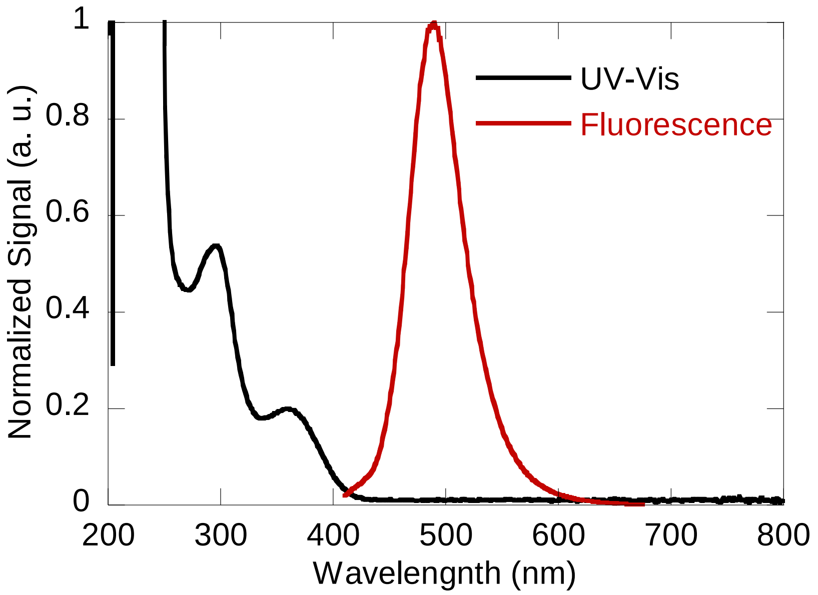

Figure S11. UV-Vis absorption and fluorescence spectra for OMe-DETC in THF solution.

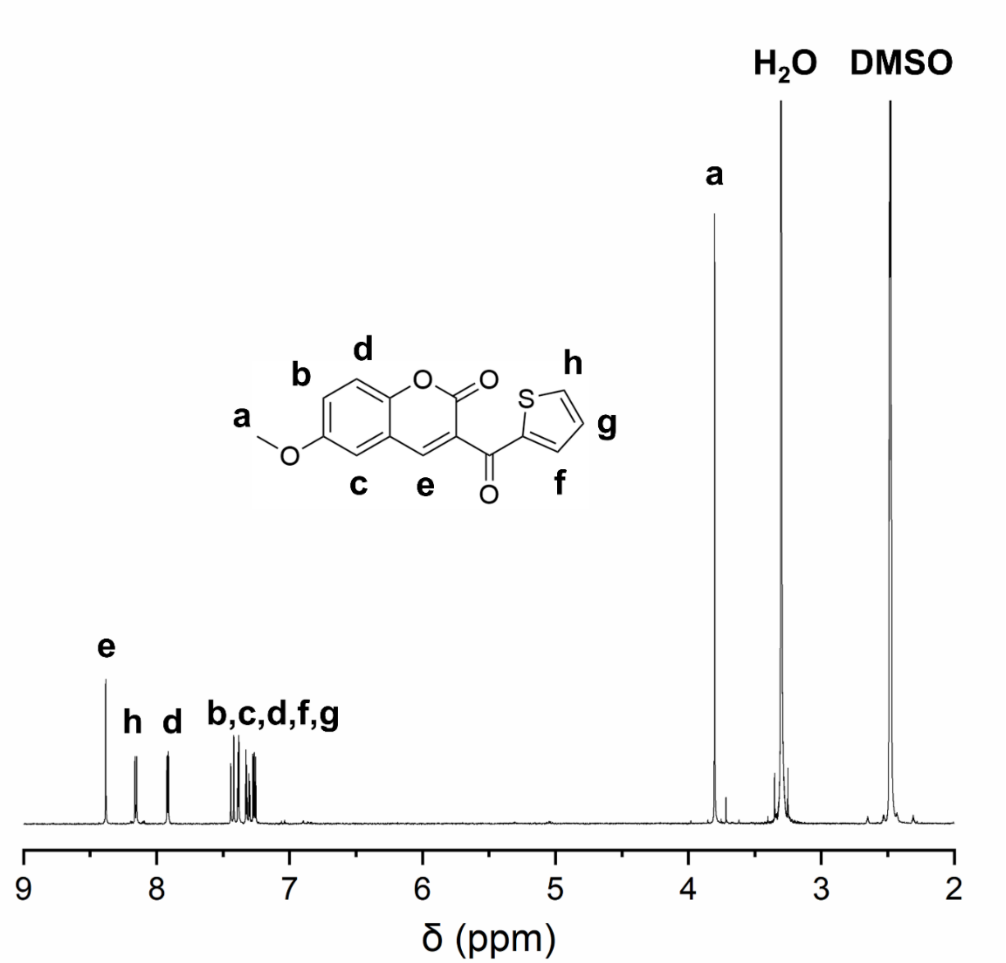

Figure S12. ^{1}H NMR spectra of 6-methoxy-3-(thiophene-2-carbonyl)-2H-chromen-2-one (DETC-OMe). Product was dissolved in deuterated dimethyl sulfoxide (DMSO-d_6).

<u>**References**</u>

[1] B. Harke, W. Dallari, G. Grancini, D. Fazzi, F. Brandi, A. Petrozza, and A. Diaspro, *Polymerization Inhibition by Triplet State Absorption for Nanoscale Lithography*, Adv. Mater. **25**, 904 (2013).

[2] P. Kiefer, V. Hahn, M. Nardi, L. Yang, E. Blasco, C. Barner-Kowollik, and M. Wegener, *Sensitive Photoresists for Rapid Multiphoton 3D Laser Micro- and Nanoprinting*, Adv. Opt. Mater. **8**, 1 (2020).

[3] T. Chi, P. Somers, D. A. Wilcox, A. J. Schuman, J. E. Johnson, Z. Liang, L. Pan, X. Xu, and B. W. Boudouris, *Substituted Thioxanthone-Based Photoinitiators for Efficient Two-Photon Direct Laser Writing Polymerization with Two-Color Resolution*, ACS Appl. Polym. Mater. **3**, 1426 (2021).

[4] I. Sakellari, E. Kabouraki, D. Gray, V. Purlys, C. Fotakis, A. Pikulin, N. Bityurin, M. Vamvakaki, and M. Farsari, *Diffusion-Assisted High-Resolution Direct Femtosecond Laser Writing*, ACS Nano **6**, 2302 (2012).
